# Supplementary material for: Knowledge and attitude of dental school faculties towards stem cell therapies and their applications
Source: PeerJ. 2025 Mar 31;13:e19127. doi: 10.7717/peerj.19127 (PMC11967409; doi:10.7717/peerj.19127)
Supplement: Supplemental Information 1 [file peerj-13-19127-s001.docx]

TableS1: Distribution of study subject’s responses towards the knowledge items of stem cell therapies and their applications

| Type of Questions | No. of Correct answers (%) | No. of wrong answers (%) | No. of not sure answers (%) |
| --- | --- | --- | --- |
| Questions about definitions | | | |
| Q1 (defined as undifferentiated stem cells) | **82 (80.4)** | 3(2.9) | 17 (16.7) |
| Q2 (divided into embryonic and adult) | **65 (63.7)** | 5(4.9) | 32 (31.4) |
| Q3 (Adult cells have same differentiation capacity as embryonic) | 36 (35.3) | 23(22.5) | **43 (42.2)** |
| Questions about Stem cell sources | | | |
| Q1 isolated from primary teeth | **59(57.8)** | 6 (5.9) | 37 (36.3) |
| Q2 isolated from pulp of permanent teeth | **56(54.9)** | 8 (7.8) | 38 (37.3) |
| Q3 Isolated from apical papilla | 36(35.3) | 13 (12.7) | **53 (52.0)** |
| Q4 isolated from apical granuloma | 28(27.5) | 21 (20.6) | **53 (52.0)** |
| Q5 Isolated from periodontal tissues | **53(52.0)** | 5(4.9) | 44 (43.1) |
| Q6 isolated from tooth follicle | **58 (56.9)** | 3(2.9) | 41(40.2) |
| Questions about stem cell applications | | | |
| Q1 have non dental applications | **51(50.0)** | 9 (8.8) | 42 (41.2) |
| Q2 utilized for regeneration of enamel | 24(23.5) | 33(32.4) | **45(44.1)** |
| Q3 Utilized for regeneration of dentin | 41(40.2) | 10(9.8) | **51(50.0)** |
| Q4 Utilized for regeneration of cementum | 7(6.9) | 43(42.2) | **52(51.0)** |
| Q5 Utilized for regeneration of pulp | **57(55.9)** | 4(3.9) | 41(40.2) |
| Q6 utilized for regeneration of periodontal structures | **53(52.0)** | 2(2.0) | 47(46.1) |
| Questions related awareness of stem cell research and applications in Saudi Arabia | | | |
| Q1 At least 3 stem cell units are available in Riyadh | 19(19.6%) | 1(1.0) | **82(80.4)** |
| Q2 Research on Embryonic stem cells are conducted in Saudi Arabia | 30(29.4) | 5(4.9) | **67(65.7)** |
| Q3 Stem cell therapy is already applied for certain leukemia treatments in Saudi Arabia | 30(29.4) | 3(2.9) | **69(67.6)** |

The highest percentage is **highlighted**
