# Supplementary material for: Knowledge and attitude of dental school faculties towards stem cell therapies and their applications
Source: PeerJ. 2025 Mar 31;13:e19127. doi: 10.7717/peerj.19127 (PMC11967409; doi:10.7717/peerj.19127)
Supplement: Supplemental Information 2 [file peerj-13-19127-s002.docx]

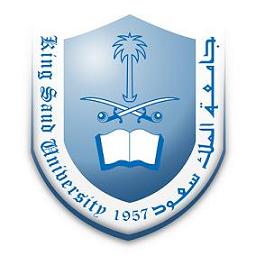


Knowledge and Attitude of Dental School Faculties in King Saud University Towards Stem Cell Therapies and Their Applications.

The following questionnaire is intended to evaluate the stem cell-based knowledge and attitudes among faculty members in collage of dentistry, KSU. It will include some demographic data, questions evaluating knowledge about the subject, attitudes towards its teaching and research participation.

You are kindly invited voluntarily fill the following questionnaire, which is expected to take less than 10 minutes to fill. We ensure a professional management of the obtained data.

Thank you

- Age:
  - 24-30
  - 30-35
  - 35-40
  - >40
- Gender:
  - Male
  - Female
- Nationality:
  - Saudi
  - Non Saudi (Please specify) ……….
- Specialty:
  - Maxillofacial surgeon
  - Oral Diagnosis/medicine
  - Endodontist/Restorative dentist
  - Periodontist/Community dentistry
  - Prosthodontist
  - Pedodontist/Orthodontist
  - Other (Please Specify) …………………
- How do you describe your stem cell-based knowledge:
  - Barely heard about stem cells
  - Basic knowledge only
  - Have good amount of knowledge with Degree/specialty/ experience in the field
- Where did you get your stem cell-based information from (more than one choice is possible)
  - Undergraduate study
  - Post graduate study
  - Mass media
  - Internet search
  - Books
  - Journal articles
  - Conferences/seminars/workshops
  - Special courses
  - Training/Researc
- Please mark (✔) the selected box:

|  | Yes | No | Not Sure |
| --- | --- | --- | --- |
| Stem cell are undifferentiated cells |  |  |  |
| Stem cells are divided into: Embryonic and Adult stem cells |  |  |  |
| Adult stem cells have the same differentiation capacity as embryonic cells |  |  |  |
| Dental stem cells have several non dental applications |  |  |  |
| Dental stem cells were successfully isolated from |  |  |  |
| - Pulp of primary teeth |  |  |  |
| - Pulp of permanent teeth |  |  |  |
| - Apical papilla |  |  |  |
| - Apical granuloma |  |  |  |
| - Periodontal tissue |  |  |  |
| - Tooth follicle |  |  |  |
| Dental stem cells were successfully utilized for regeneration of: |  |  |  |
| - Enamel |  |  |  |
| - Dentin |  |  |  |
| - Cementum |  |  |  |
| - Pulp |  |  |  |
| - Periodontal structures |  |  |  |

- Please mark (✔) the selected box Regarding stem cells in Saudi Arabia

|  | Yes | No | Not Sure |
| --- | --- | --- | --- |
| Stem cell are undifferentiated cells |  |  |  |
| Stem cells are divided into: Embryonic and Adult stem cells |  |  |  |
| Adult stem cells have the same differentiation capacity as embryonic cells |  |  |  |
| Dental stem cells have several non dental applications |  |  |  |
| Dental stem cells were successfully isolated from |  |  |  |
| - Pulp of primary teeth |  |  |  |
| - Pulp of permanent teeth |  |  |  |
| - Apical papilla |  |  |  |

|  | Yes | No | Not Sure |
| --- | --- | --- | --- |
| At least 3 stem cell units are available in Riyadh |  |  |  |
| Research on Embryonic stem cells are conducted in Saudi Arabia |  |  |  |
| Stem cell therapy is already applied for certain leukemia treatments in Saudi Arabia |  |  |  |

- Do any of your lectures contain stem cell-based information?
  - Yes: Please specify
    - Under graduate courses
    - Post graduate courses
    - Both
  - No
- What do you think about stem cell education in King Saud University?

|  | Strongly agree | Agree | Disagree | Strongly disagree |
| --- | --- | --- | --- | --- |
| Stem cell therapy should be included in student’s **undergraduate** courses |  |  |  |  |
| Stem cell therapy should be included into dental **postgraduate** courses |  |  |  |  |
| Stem cell lectures should be provided as **extracurricular activities** |  |  |  |  |

- Are you interested in participation in stem cell-based research?
  - Yes
  - No
  - Not Sure
- If you have removed/extracted any body tissue for medical reasons, would you agree to freely donate it for stem cell research?
  - Yes
  - No
  - Not sure
- In your opinion, what are the possible barriers for performing stem cell-based research?

|  | Strongly agree | Agree | Disagree | Strongly disagree |
| --- | --- | --- | --- | --- |
| Difficulties in accessibility to research center |  |  |  |  |
| Lack of proper facilities |  |  |  |  |
| Lack of well trained technicians |  |  |  |  |
| Difficulty of obtaining materials |  |  |  |  |
| Time limitations |  |  |  |  |
| High Expenses |  |  |  |  |
| Ethical/ religious issues |  |  |  |  |
| The need for different type of training |  |  |  |  |
| Lack of interest |  |  |  |  |

Thank you
