# Supplementary material for: Knowledge and attitude of dental school faculties towards stem cell therapies and their applications
Source: PeerJ. 2025 Mar 31;13:e19127. doi: 10.7717/peerj.19127 (PMC11967409; doi:10.7717/peerj.19127)
Supplement: Supplemental Information 3 [file peerj-13-19127-s003.docx]

Table.1: Distribution of socio-demographic professional characteristics of study subjects (n=102)

| Characteristics | No. |
| --- | --- |
| Age groups (in years)  24-30  31-35  36-40  >40  Gender  Male  Female  Nationality  Saudi  Non-Saudi  Specialty  Maxillofacial surgery  Oral Diagnosis  Endodontist & Restorative dentistry  Periodontist & Community dentistry  Prosthodontist  Pedodontist & Orthodontist  Others | 1  2  3  4  1  2  1  2  1  2  3  4  5  6  7 |

Table.2: Distribution of study subject’s responses towards the knowledge items of Stem Cell Therapies and their applications (actual responses)

| Knowledge items | Responses- No. (%) | | |
| --- | --- | --- | --- |
|  | Yes | No | Not sure |
| Stem cell are undifferentiated cells  Stem cells are divided into: Embryonic and Adult stem cells  Adult stem cells have the same differentiation capacity as embryonic cells  Dental stem cells have several non- dental applications  Dental stem cells were successfully isolated from:  • Pulp of primary teeth  • Pulp of permanent teeth  • Apical papilla  • Apical granuloma  • Periodontal tissue  • Tooth follicle  Dental stem cells were successfully utilized for regeneration of:  • Enamel  • Dentin  • Cementum  • Pulp  • Periodontal structures  At least 3 stem cell units are available in Riyadh  Research on Embryonic stem cells are conducted in Saudi Arabia  Stem cell therapy is already applied for certain leukemia treatments in Saudi Arabia | 1  1  1  1  1  1  1  1  1  1  1  1  1  1  1  1  1  1 | 2  2  2  2  2  2  2  2  2  2  2  2  2  2  2  2  2  2 | 3  3  3  3  3  3  3  3  3  3  3  3  3  3  3  3  3  3 |

Table.3: Distribution of study subject’s responses towards the knowledge items of Stem Cell Therapies and their applications (corrected answers)

| Knowledge items | Responses- No. (%) | | |
| --- | --- | --- | --- |
|  | Yes | No | Not sure |
| Stem cell are undifferentiated cells  Stem cells are divided into: Embryonic and Adult stem cells  Adult stem cells have the same differentiation capacity as embryonic cells  Dental stem cells have several non- dental applications  Dental stem cells were successfully isolated from:  • Pulp of primary teeth  • Pulp of permanent teeth  • Apical papilla  • Apical granuloma  • Periodontal tissue  • Tooth follicle  Dental stem cells were successfully utilized for regeneration of:  • Enamel  • Dentin  • Cementum  • Pulp  • Periodontal structures  At least 3 stem cell units are available in Riyadh  Research on Embryonic stem cells are conducted in Saudi Arabia  Stem cell therapy is already applied for certain leukemia treatments in Saudi Arabia | 2  2  0  2  2  2  2  0  2  2  0  2  0  2  2  2  2  2 | 0  0  2  0  0  0  0  2  0  0  2  0  2  0  0  0  0  0 | 1  1  1  1  1  1  1  1  1  1  1  1  1  1  1  1  1  1 |

Table.4: Distribution of study subject’s responses towards the sources of Stem cell information, Stem cell education, research, practice & self-description of stem cell-based knowledge

| Items for source of information, education, research, practice & self-description | No. (%) |
| --- | --- |
| Academic source  Under graduate study  Post graduate study  Mass media  Internet search  Books  Journal articles  Conferences/seminars/workshops  Special courses  Training/Research  Any lectures contain stem cell information  Yes-Under graduate courses  Yes-Post graduate courses  No  Stem cell therapy should be included in student’s undergraduate courses  Strongly agree  Agree  Disagree  Strongly disagree  Stem cell therapy should be included into dental post graduate courses  Strongly agree  Agree  Disagree  Strongly disagree  Stem cell lectures should be provided as extra-curricular activities  Strongly agree  Agree  Disagree  Strongly disagree  Interested in participation in stem cell-based research  Yes  No  Not sure  If you have removed/extracted any body tissue for medical reasons, would you agree to freely donate it for stem cell research?  Yes  No  Not sure  How do you describe your stem cell- based Knowledge?  Barely heard about stem cells  Basic knowledge only  Have good amount of knowledge, with a Degree/specialty/ experience in the field | 1  1  1  1  1  1  1  1  1  1  2  0  1  2  3  4  1  2  3  4  1  2  3  4  1  2  3  1  2  3  1  2  3 |

Table.5: Distribution of ranking of responses towards the possible barriers for performing Stem cell-based research

| Barriers | Responses | | | | Ranking of barrier |
| --- | --- | --- | --- | --- | --- |
|  | Strongly agree | Agree | Disagree | Strongly  disagree |  |
| Difficulties in accessibility to research center | 1 | 2 | 3 | 4 |  |
| Lack of proper facilities | 1 | 2 | 3 | 4 |  |
| Lack of well -trained technicians | 1 | 2 | 3 | 4 |  |
| Difficulty of obtaining materials | 1 | 2 | 3 | 4 |  |
| Time limitations | 1 | 2 | 3 | 4 |  |
| High Expenses | 1 | 2 | 3 | 4 |  |
| Ethical/ religious issues | 1 | 2 | 3 | 4 |  |
| The need for different type of training | 1 | 2 | 3 | 4 |  |
| Lack of interest | 1 | 2 | 3 | 4 |  |
